# Interlukin-4 weakens resistance to stress injury and megakaryocytic differentiation of hematopoietic stem cells by inhibiting *Psmd13* expression

**DOI:** 10.1038/s41598-023-41479-6

**Published:** 2023-08-31

**Authors:** Ai Gao, Shuhui Xu, Qing Li, Caiying Zhu, Fengjiao Wang, Yajie Wang, Sha Hao, Fang Dong, Hui Cheng, Tao Cheng, Yuemin Gong

**Affiliations:** 1 State Key Laboratory of Experimental Hematology, Tianjin, China; 2 Institute of Hematology and Blood Disease Hospital, Chinese Academy of Medical Sciences and Peking Union Medical College, Tianjin, China; 3 Department of Hematology, The First Affiliated Hospital of Nanjing Medical University, Jiangsu Province Hospital, Nanjing, China; 4 Department of Medical Oncology, Tianjin Medical University General Hospital, Tianjin, China; 5 Center for Stem Cell Medicine, Chinese Academy of Medical Sciences, Tianjin, China; 6 Department of Stem Cell and Regenerative Medicine, Peking Union Medical College, Tianjin, China; 7 Medical School, Kunming University of Science and Technology, The First People’s Hospital of Yunnan Province, Kunming, China

**Keywords:** Stress signalling, Growth factor signalling

## Abstract

Thrombocytopenia is a major and fatal complication in patients with acute myeloid leukemia (AML), which results from disrupted megakaryopoiesis by leukemic niche and blasts. Our previous research revealed that elevated interleukin-4 (IL-4) in AML bone marrow had adverse impact on multiple stages throughout megakaryopoiesis including hematopoietic stem cells (HSCs), but the specific mechanism remains unknown. In the present study, we performed single-cell transcriptome analysis and discovered activated oxidative stress pathway and apoptosis pathway in IL-4Rα^high^ versus IL-4Rα^low^ HSCs. IL-4 stimulation in vitro led to apoptosis of HSCs and down-regulation of megakaryocyte-associated transcription factors. Functional assays displayed higher susceptibility of IL-4Rα^high^ HSCs to tunicamycin and irradiation-induced apoptosis, demonstrating their vulnerability to endoplasmic reticulum (ER) stress injury. To clarify the downstream signaling of IL-4, we analyzed the transcriptomes of HSCs from AML bone marrow and found a remarkable down-regulation of the proteasome component *Psmd13,* whose expression was required for megakaryocytic-erythroid development but could be inhibited by IL-4 in vitro*.* We knocked down *Psmd13* by shRNA in HSCs, and found their repopulating capacity and megakaryocytic differentiation were severely compromised, with increased apoptosis in vivo. In summary, our study uncovered a previous unrecognized regulatory role of IL-4-*Psmd13* signaling in anti-stress and megakaryocytic differentiation capability of HSCs.

## Introduction

The main causes of death, other than resistant disease or relapse, in patients with acute myeloid leukemia (AML) are hemorrhage and infection, stemming from severe thrombocytopenia and neutropenia^1^. Studies have shown that a variety of cells, cytokines, exosome contents are tampered in the leukemic microenvironment to support leukemia progression while inhibit proliferation and differentiation of residual hematopoietic stem and progenitor cells (HSPCs)^2–8^. Our previous work uncovered a link between hyperactive Interlukin-4 (IL-4) signaling and defective megakaryopoiesis in AML bone marrow. IL-4 exerted inhibitory effects on multiple stages along the path of megakaryocyte (MK) differentiation, from hematopoietic stem cells (HSCs) to MK progenitors. IL-4 antibody in combination with induction chemotherapy alleviated thrombocytopenia and prolonged overall survival of AML mice^6^. Yet the regulatory effect of IL-4 on HSCs and its mechanism remain unclear.

In the present study, we display that IL-4 renders HSCs susceptible to endoplasmic reticulum (ER) stress-induced apoptosis. IL-4 inhibits expression of the proteasome component *Psmd13,* whose down-regulation is detected in HSCs from AML bone marrow and impairs their repopulating capacity and megakaryocytic differentiation after transplantation.

## Methods

### Mice

C57BL/6-Ly5.1 (Ly5.1) and C57BL/6-Ly5.2 (Ly5.2) were purchased from the State Key Laboratory of Experimental Hematology (SKLEH). R26-tdTomato mice^9^ were purchased from Jackson Lab. β-actin-eGFP mice^10^ were kindly provided by Bing Liu (Academy of Military Medical Sciences, Beijing, China). Pf4-Cre mice^11^ were a gift from Junling Liu (Shanghai Key Laboratory of Tumor Microenvironment and Inflammation, China). Pf4-Cre mice were bred with R26-tdTomato mice to obtain Pf4-Tdtomato reporter mice. Mice experiments were approved by Institutional Animal Care and Use Committee of SKLEH and were carried out in accordance with ARRIVE guidelines^12^.

### Flow cytometry

Detailed staining and enrichment procedures for flow cytometry have been previously described^13^.

Cell surface markers for phenotypical analyses of hematopoietic cells are listed as following: LT-HSC (Lin-cKit + Sca1 + Flt3-CD34-), MPP (Lin-cKit + Sca1 + Flt3 + CD34 +), MK (SSC^high^CD41^high^).

Antibodies used are listed in Supplementary Table S1.

For apoptosis analysis, cells were cultured with mIL-4 (10 ng/mL) for 24 h and labeled with surface markers, then stained with an Annexin-V antibody and 7-AAD in binding buffer according to the manufacturer’s instructions (BD Bioscience).

### Colony-forming assays

Cells (800 LKS + cells/well for multi-lineage colony formation in 24-well plates in a 0.5 mL volume) were directly pipetted into M3434 methylcellulose (Stem Cell Technologies) supplemented with 10 ng/mL mTPO and 10 ng/mL mFlt3L and incubated at 37 °C for 7–10 days in a humidified chamber.

### Single cell liquid culture

For multi-lineage potential of HSCs, single-cell sorted HSCs were grown in IMDM (Gibco) supplemented with 10% FBS, L-Gln (2 mM), 1% β-mercaptoethanol (50 μM), mSCF (10 ng/mL), mTPO (10 ng/mL), mIL-3 (10 ng/mL), hEPO (1 U/mL), mGM-CSF (10 ng/mL). Colonies were evaluated after 9 days of culture. Unless otherwise indicated, cytokines were obtained from PeproTech.

### Virus transduction

Production and packaging of lentivirus for stably knocking down *Psmd13* (hU6-MCS-Ubiquitin-EGFP-IRES-puromycin) were accomplished by the company (Shanghai Genechem Co., LTD). LKS+ cells (CD45.2+) were flow sorted from donor mice. Cells were pre-cultured for 1 day, then transduced with viruses and incubated for another 3 days. The culture was maintained in StemSpan SFEM medium containing 100 ng/mL mSCF and 100 ng/mL mTPO. After transduction, GFP+ cells were sorted for assays.

### Transplantation

For measurement of the reconstitution activity, IL4Rα^high^ and IL4Rα^low^ HSCs were sorted from the BM of β-actin-GFP mice, and 1700 cells per group were then transplanted into lethally irradiated (9.5 Gy) B6-Ly5.2 recipient mice in competition with 3 × 105 CD45.2 + BM cells.

For *Psmd13* knockdown assay, GFP + transduced cells were transplanted together with 3 × 10^5^ CD45.2 + BM cells into lethally irradiated B6-Ly5.2 mice.

### Single-cell RNA sequencing

LT-HSCs were purified by FACS sorting. Each single LT-HSC was placed into the lysis buffer by mouth pipette. Library preparation and processing of single cell RNA-seq data followed the detailed procedure described in Li et al.’s study^14^. Unique molecular identifiers (UMIs) was used to directly measure the number of transcripts for each gene. Cells with at least 2000 genes and more than 100,000 transcripts detected, and more than 40% reads mapping to genome were remained for further analysis. For all 34 single cells, 30 single cells passed quality control.

Differential expression genes between IL4Rα^high^ and IL4Rα^low^ HSCs were identified by FindMarkers function of Seurat (Version 2.0)^15^ with default parameters. Log2 (TPM/10 + 1) was used as expression values. Genes with log2(fold-change) ≥ 0.59 or ≤ − 0.59 and p value < 0.05 were selected as differential expressed genes^15^. Canonical pathway analyses were performed by Ingenuity Pathway Analysis (QIAGEN Bioinformatics).

### UMI-mRNA sequencing

LKS + cells were purified by FACS sorting. Total RNA was used as the input material for the library preparation. mRNA was firstly captured using mRNA Capture Beads with Oligo(dT), and purified with Binding Buffer and Washing Buffer. mRNA was then randomly fragmented into 100–200 nt in the Fragmentation Buffer and reverse-transcribed into cDNA subsequently. Synthesized cDNA was reclaimed using DNA Clean Beads and subjected to the adaptor ligation step. Purified cDNA was ligated to the pre-mixed adaptors with UMI using Ligase and Ligase Buffer in a one-step PCR reaction. The ligation products were again purified using DNA Clean Beads and then amplified using PCR. PCR products were finally purified using DNA Clean Beads and reclaimed with nuclease-free H_2_O. After the library preparation and pooling of different samples, the samples were subjected for Illumina sequencing. The libraries were sequenced on Illumina novaseq 6000 Platform for 6G raw data, and generated 150 nt pair-end reads.

### Statistics

Unless otherwise stated, data are expressed as mean ± standard error of the mean (SD) as indicated. P values were generated using unpaired Student’s t-test and analysis of variance. GraphPad Prism 5.0 software was used for the statistical analysis.

### Ethical approval

All mice were bred and maintained under specific pathogen–free conditions in the animal facility at the Institute of Hematology in accordance with Institutional Animal Care and Use Committee of the Institute of Hematology.

## Results

### IL-4 stimulation increased apoptosis of HSCs

**Figure 1:**
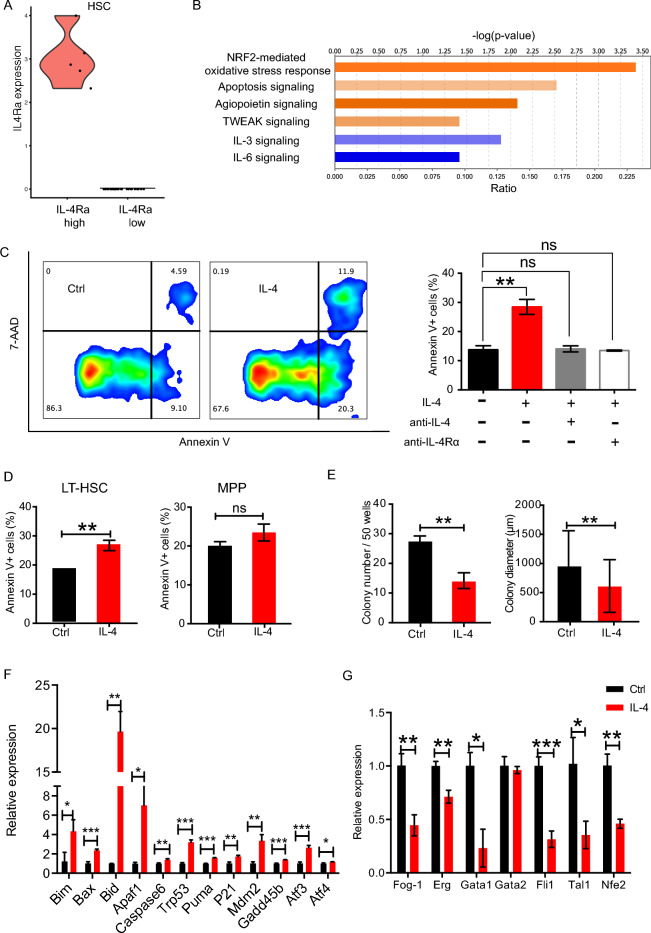
Increased apoptosis of HSCs in response to IL-4. (**A**) Violin plots showing distribution of expression levels of IL-4Ra across single cells of LT-HSCs. N = 5 for IL-4Rahigh group and n = 25 for IL4Ralow group. (**B**) Predicted activation and inhibition of canonical pathways in IL-4Rahigh vs IL-4Ralow HSCs by Ingenuity Pathway Analysis (IPA). (**C**) Left, representative FACS plots for apoptosis analysis of LKS+ cells with and without mIL-4 (10 ng/mL) treatment for 24 h. Right, the apoptotic rate of LKS+ cells upon indicated treatments for 24 h. Anti-IL-4 and anti-IL-4Rα antibody were used both at a concentration of 10 μg/mL. N = 3 per group. 3 independent experiments. (**D**) Apoptosis analysis of LT-HSCs and MPPs in the absence or presence of mIL-4 (10 ng/mL) for 24 h. (**E**) Single-cell colony number and diameter of LT-HSCs after 9 days liquid culture in the absence or presence of mIL-4 (10 ng/mL) (n = 60 cells per group). (**F**) Expression of apoptosis and stress response associated genes quantified by qRT-PCR (normalized to Gapdh) in LKS+ cells with (red bar) or without (black bar) IL-4 treatment for 1 h. N = 3 per group. (**G**) Megakaryocytic transcription factors expression quantified by qRT-PCR (normalized to Gapdh) in LKS + cells cultured in vitro with or without IL-4 (10 ng/mL) for 24 h. N = 3 per group. Data are shown as mean ± SD. *p < 0.05, **p < 0.01, ***p < 0.001. *ns* no significance.

To search for clues to the innate effect of endogenous IL-4 on HSCs in steady state, we performed single-cell RNA sequencing (scRNA-seq) and acquired transcriptomes of 30 long-term HSCs (LT-HSCs) finally after strict quality control. We divided these transcriptomes into IL-4Rα^high^ and IL-4Rα^low^ groups based on their expression of IL-4Rα beyond or below detection threshold respectively (Fig. 1A), hypothesizing that IL-4Rα^high^ HSCs might exhibit more remarkable IL-4-primed gene expression profiling compared with IL-4Rα^low^ HSCs during their development in vivo. Ingenuity Pathway Analysis (IPA) of differentially expressed genes between two groups revealed that four pathways were possibly activated (p < 0.05, z-score > 0) whereas a large majority of canonical pathways were possibly inhibited (z-score > 0) (Supplementary Table S1). Among the activated pathways, NRF2-mediated oxidative stress pathway and apoptosis pathway were top two enriched (Fig. 1B). As expected, we observed significantly increased apoptotic rate of Lin-c-Kit + Sca1+ (LKS+) cells 24 h after IL-4 exposure (Fig. 1C). And the pro-apoptotic effect of IL-4 could be completely abrogated by IL-4 neutralizing antibody and anti-IL4Rα antibody (Fig. 1C). Our previous results showed that HSC-enriched LKS+ cells exhibted higher IL-4Rα level and more prominent activation of pStat6 signaling in response to exogenous IL-4 stimulation, compared to myeloid progenitors^17^. Among LKS+ subpopulations, LT-HSCs (CD34−Flt3−) were more susceptible to IL4-induced apoptosis than MPPs (CD34 + Flt3+) (Fig. 1D). Single cell liquid culture of LT-HSCs yielded significantly fewer and smaller colonies with IL-4 than without (Fig. 1E). Similarly, cell culture of LKS+ cells showed that the absolute cell number with IL-4 decreased compared with those without IL-4 (Supplementary Fig. 1C). Then, we extended the cell cycle analysis to the LKS+ cells 24 h after IL-4 exposure to determine whether IL-4 changed the proliferation or cell cycle state. However, Ki67 staining showed no significant difference (Supplementary Fig. 1A,B). We detected prominent up-regulation of pro-apoptotic genes (*Bim*, *Bax*, *Bak1*, *Bid*, *Apaf1*, *Caspase6*), p53 signaling genes (*Trp53*, *Puma*, *p21*, *Mdm2*) and stress-response genes (*Atf3*, *Gadd45b*, *Atf4*) in LKS+ cells within 1 h after IL-4 exposure (Fig. 1F). Among them, *Caspase6* and *Atf3* had been reported to be directly regulated by IL-4/Stat6 in lymphocytes^18^ and to facilitate the process of apoptosis^19–21^. Besides, megakaryocyte-associated transcription factors except for Gata2 were universally inhibited in LKS+ cells after IL-4 treatment (Fig. 1G), indicating reduced megakaryocytic differentiation potential.

### IL-4Rα^high^ HSCs were more susceptible to endoplasmic reticulum stress induced apoptosis

**Figure 2:**
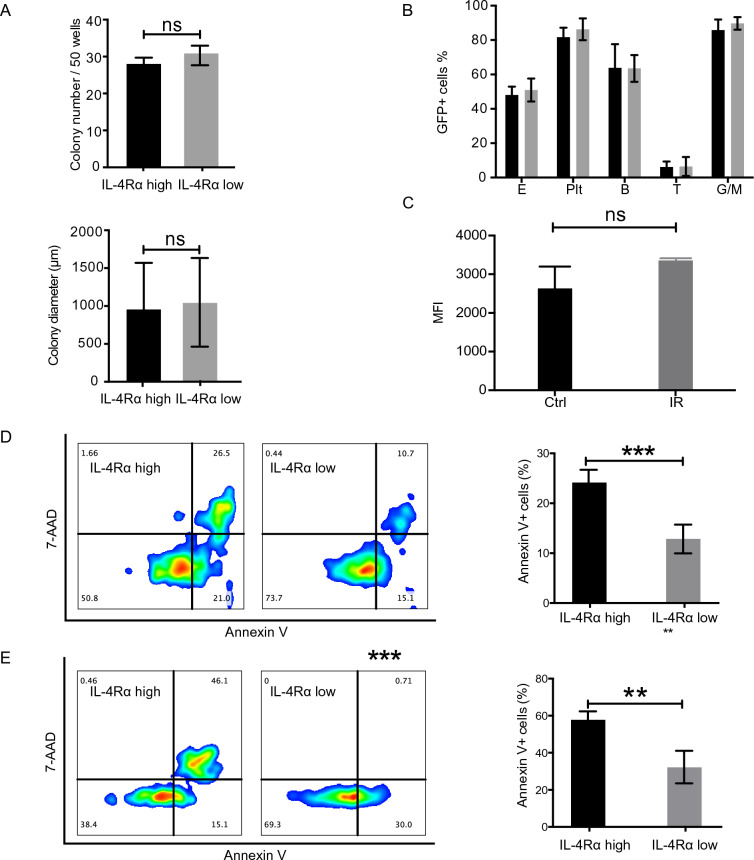
Susceptibility of IL4Rαhigh HSCs to unfolded protein response (UPR) and irradiation induced apoptosis. (**A**) Single-cell colony number and diameter of IL4Rαhigh and IL4Rαlow LT-HSCs after 9 days liquid culture (n = 50 cells per group). (**B**) Mean contribution of 1700 IL-4Rαhigh (black bar) and IL-4Rαlow (grey bar) LT-HSCs to myeloid cells, T cells, B cells, platelets and erythrocytes in peripheral blood 12 weeks after transplantation. N = 7 mice per group. 2 independent experiments. (**C**) Mean fluorescence intensity (MFI) of IL-4Rα in LT-HSCs 16 h post 2 Gy radiation. N = 3 mice per group. 2 independent experiments. (**D**) The apoptotic rate of FACS-sorted IL-4Rαhigh and IL-4Rαlow LT-HSCs after treated with tunicamycin (0.35 μg/mL) for 24 h. N = 4–6 for each group. (E) The apoptotic rate of IL-4Rαhigh and IL-4Rαlow LT-HSCs 16 h post 2 Gy radiation. N = 3 mice per group. 2 independent experiments. Data are shown as mean ± SD. **p < 0.01, ***p < 0.001. *ns* no significance.

As the transcriptomes of IL-4Rα^high^ HSCs displayed apoptosis signature and reduced response to growth stimulatory signaling such as IL-3 and IL-6 (Fig. 1B), we expected them to exhibit a growth disadvantage compared with IL-4Rα^low^ HSCs. However, single cell colony assay did not demonstrate significant difference in colony number or size between IL-4Rα^high^ and IL-4Rα^low^ HSCs (Fig. 2A). Besides, IL-4Rα^high^ and IL-4Rα^low^ HSCs did not show significant difference in their reconstitution capacity of all five lineages including erythrocytes and platelets in transplantation assays using β-actin-GFP reporter mice as donors (Fig. 2B). We then investigated their response to stress conditions by treating them in vitro with tunicamycin, an inhibitor of protein glycosylation that induced unfolded protein response (UPR) and endoplasmic reticulum (ER) stress^22,23^. As a result, significantly higher apoptotic rate was detected in IL-4Rα^high^ group than IL-4Rα^low^ group, indicating that IL-4Rα^high^ HSCs were more susceptible to ER stress induced apoptosis (Supplementary Fig. 2A; Fig. 2D).

Irradiation causes DNA damage and prolonged elevation of reactive oxygen species (ROS) in HSCs^24–26^ and triggers ER stress to restore homeostasis or initiate apoptosis^27^. We exposed mice to 2 Gy irradiation and examined the apoptotic rate of HSC subpopulations 16 h later. We detected the change in IL-4R expression on HSCs before and after irradiation, but there was no significant difference (Fig. 2C). As the result showed, greatly larger proportions of IL-4Rα^low^ HSCs remained viable than IL-4Rα^high^ HSCs, suggesting that IL-4Rα^low^ HSCs were more resistance to irradiation-induced stress (Supplementary Fig. 1B; Fig. 2E).

To gain further insight into the role of IL-4 on HSC maintenance, we isolated LKS+ cells from healthy BM and treated with IL-4 or not for 24 h for RNA-seq assay (Supplementary Fig. 2A,B). Gene expression data showed downregulation of hematopoietic stem progenitor cell differentiation genes and megkaryocyte differentiation genes in LKS+ cells treated with IL-4 (Supplementary Fig. 2C), implying IL-4 inhibited megakaryocytic differentiation capability of HSCs. In addition, apoptosis-associated genes and response to endoplasmic reticulum stress genes were positively enriched in LKS+ cells treated with IL-4 (Supplementary Fig. 2C). These data indicated that IL-4 signaling is related to UPR and ER stress, suggesting that IL-4 may act possibly as a stress signaling in HSCs.

### IL-4 decreases *Psmd13* expression in HSCs

**Figure 3:**
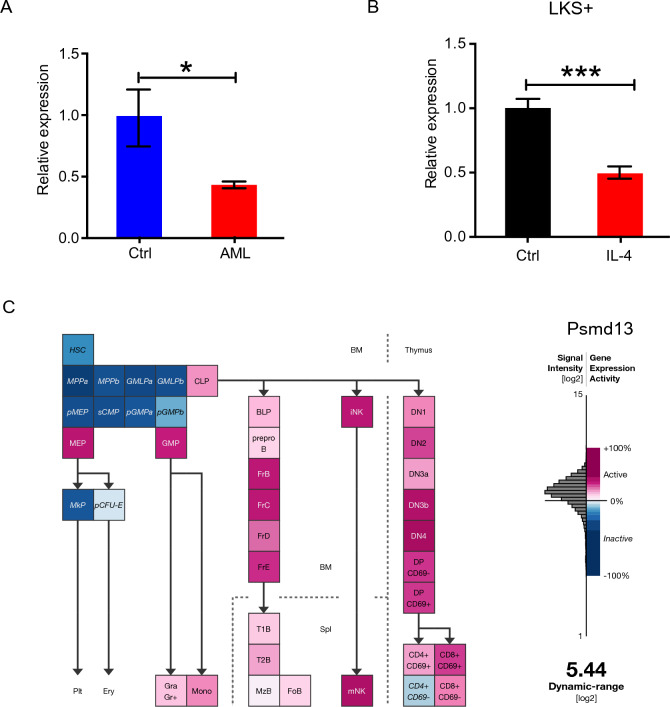
IL-4 induces Psmd13 down-regulation. (**A**) qRT-PCR of the relative expression of Psmd13 in LKS+ cells from AML and control mice (n = 3 mice per group). (**B**) Histograms showing Psmd13 mRNA expression in LKS + cells with or without mIL-4 (10 ng/mL) and anti-IL4 (10 μg/mL) treatment for 24 h. N = 3 per group. (**C**) The gene expression profile of Psmd13 in HSPC subsets in ‘‘Gene Expression Commons’’ (https://gexc.stanford.edu). Calculated low/high expression level was represented by a color-coded heatmap: red represents high expression, white represents threshold level expression, and blue represents low expression. Data are shown as mean ± SD. *p < 0.05, ***p < 0.001. *ns* no significance.

The compromised adaptation of IL-4Rα^high^ HSCs to ER stress inspired us to consider the possibility that IL-4 exerted its effect on HSCs by influencing protein metabolism. Proteasome is a major organelle responsible for degradation of redundant and misfolded proteins. The function of proteasome to lighten protein load is pivotal in UPR and ER stress response. In LKS+ cells isolated from AML mice bone marrow, we detected a significant reduced expression of *Psmd13*, a component of proteasome (Fig. 3A). Accordingly, in LKS+ cells exposed to IL-4, we observed robust inhibition of *Psmd13* expression, and the inhibition effect of IL-4 could be completely abrogated by anti-IL4Rα antibody (Fig. 3B). Interestingly, megakaryocytic-erythroid progenitors (MEPs) possessed remarkably higher expression of *Psmd13* than their upstream HSPC subsets (Fig. 3C), suggesting its possibly essential role in regulating MEP development.

### *Psmd13* down-regulation impairs megakaryocytic development

**Figure 4:**
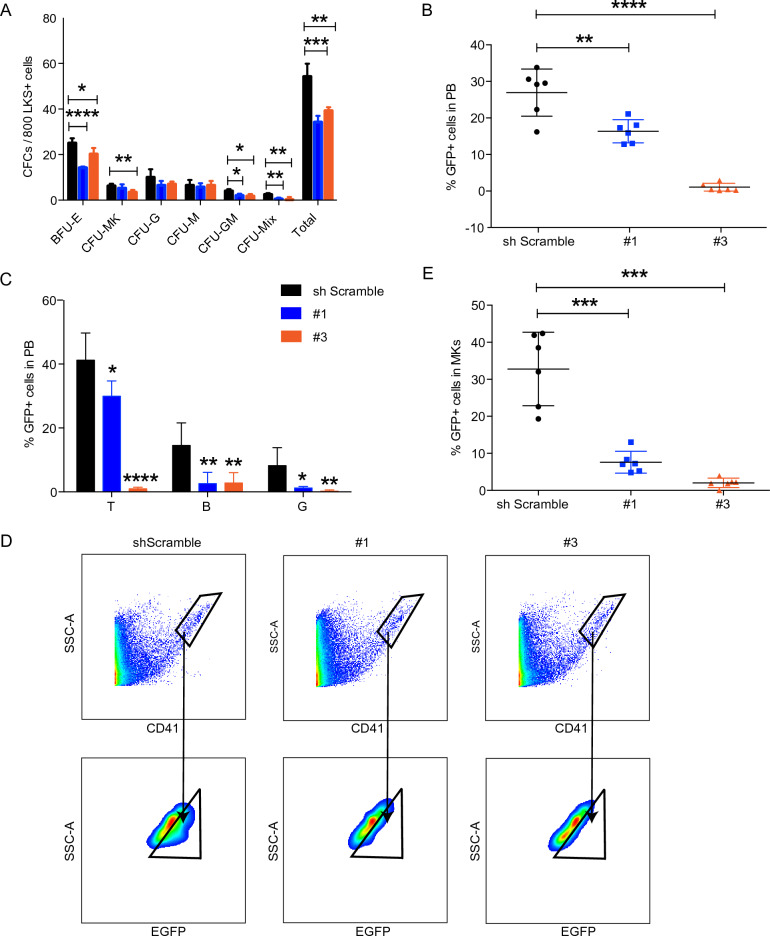
Impairment of megakaryocytic development by Psmd13 down-regulation. (**A**) In vitro colony forming ability of Psmd13 knockdown LKS+ cells. (**B**–**E**) Reconstitution status of Psmd13 shRNA (1#) or scramble control transduced LKS+ cells 6 weeks after transplantation. C, percentage of donor-derived EGFP+ cells in peripheral blood (PB). D, fractions of T, B, and myeloid cell in EGFP + PB nucleated cells. E–F, percentage of donor-derived EGFP+ cells in bone marrow megakaryocytes (MKs). N = 6–7 mice per group. 2 independent experiments. Data are shown as mean ± SD. *p < 0.05, **p < 0.01, ***p < 0.001. *ns* no significance.

According to the above results, we knocked down *Psmd13* by shRNA lentivirus in LKS+ cells (Supplementary Fig. 4A,B) and found *Psmd13* suppression inhibited clonogenic potential of LKS+ cells (Fig. 4A). We then transplanted them into lethally irradiated mice to see their capacity to reconstitute megakaryocytic lineage. After 4 weeks, *Psmd13* knockdown (KD) LKS+ cells exhibited significantly reduced overall reconstitution rate than scramble control (Fig. 4B). Among donor-derived nucleated cells in peripheral blood, we observed inhibited reconstitution of B, T, myeloid lineages and platelets compared to scramble shRNA control (Fig. 4C, Supplementary Fig. 4C,D). In bone marrow, the megakaryocytic reconstitution rate of *Psmd13* KD LKS+ cells was significantly reduced than that of scramble control (Fig. 4D,E). Collectively, *Psmd13* down-regulation impaired the repopulating potential of HSCs, especially to myeloid and megakaryocytic lineages.

Since IL-4 treatment caused apoptosis in LKS+ cells, we next investigated the status of apoptosis of *Psmd13* KD cells, to check if it is consistent with IL-4 exposure. The results showed that there was no significant difference in the proportion of apoptosis between the two KD groups and the control group (Supplementary Fig. 5A,B), which might be caused by the limitation of cell culture conditions in vitro and the short time of gene transfection. Then, according to the above transplantation scheme, we analyzed the apoptosis of donor-derived LKS+ cells four weeks after the transplantation of *Psmd13* KD LKS+ cells, and found that the apoptosis rate of KD group was significantly higher than that of the control group (Supplementary Fig. 5C,D), which was consistent with the effect of IL-4.

## Discussion

Our previous study reported overproduction of IL-4 from AML bone marrow and its inhibitory effect throughout the process of megakaryopoiesis in vivo^6^. The results suggested that IL-4 could be a promising therapeutic target combined with chemotherapy in AML, but the specific influence of IL-4 on normal HSPCs has been barely understood. In this study, we showed a weak resistance of HSCs to IL-4-induced apoptosis. Among HSCs, IL-4Ra^high^ subset was more susceptible to UPR and irradiation-induced apoptosis than IL-4Ra^low^. Stress can lead to severe functional loss or the persistence of oncogenes in HSCs, thus increasing the risk of leukemia^28,29^. We hypothesize that the interaction between immune cells-derived IL-4 and IL-4Rα on HSCs, and its pro-apoptotic consequence, may be an approach to clear away damaged or senescent HSCs after stress. Whether up-regulated IL-4Rα on HSCs is a signal calling for elimination and the underlying mechanism warrants further investigation.

A recent study from Pena-Martinez P et al. suggested the pro-apoptotic effect of IL-4 also occurred in AML blasts and it might be a potential therapeutic approach for AML^30,31^. They observed the caspase signaling pathway were significantly enriched in leukemia cells treated with IL-4 compared with the untreated group. However, we found IL-4-induced apoptosis was independent of caspase cascade in that it could not be rescued by either caspase-6 specific inhibitor or pan-caspase inhibitor (data not shown). The interrupted HSC function by IL-4-induced *Psmd13* down-regulation hinted that proteasome system might also participate in the molecular mechanism. Regulation of HSC function via ubiquitin proteasome system has been studied by deleting different members of this complicated system using genetically modified models, which yielded distinct phenotypes by influencing different substrates^32,33^. As many of these components are enzymes and relatively easy for artificial intervention as targets, more attention should be paid to the role of ubiquitin proteasome system in stress hematopoiesis. Our research is limited in the axis of IL-4-Psmd 13, and we lack direct evidence that IL-4 signaling induces ER stress and UPR in HSCs, which would be explored through Psmd 13 knockout mice in the future to continue our research.

By performing transplantation assays on LKS+ cells, *Psmd13* KD cells exhibited significantly reduced overall reconstitution rate than scramble control. Among donor-derived nucleated cells in peripheral blood, we observed inhibited reconstitution of B, T, myeloid lineages and platelets compared to scramble shRNA control, which is not restricted to Mk-lineage. To figure this out, we will perform RNA-seq assay on *Psmd13* KD LKS+  cells and scramble control in our future study, so as to clarify whether *Psmd3* KD effect is restricted to Mk-lineage from the gene level. As for RBC chimerisms, we found *Psmd13* suppression inhibited BFU-E clonogenic potential of LKS+ cells. However, in the transplantation assays, we used platelet-specific PF4-tdTomato reporter mouse LKS+ cells to infect shRNA, and indeed did not detect red RBC reconstruction, which will be perfected in our future study. Studies showed that platelet proteasome regulates platelet lifespan and viability only partially enhances platelet activation and aggregation by activating NF-κB^34^. More studies are, therefore, needed to explore how the proteasome system regulates the differentiation and development of Mk-lineage.

In summary, our study established a previous unrecognized link between IL-4-*Psmd13* signaling and anti-stress as well as megakaryocytic differentiation potential of HSCs. Our findings provided evidence to counteract IL-4 signaling and to correct the disrupted proteasome function as a possible approach to alleviate thrombocytopenia in AML.

### Supplementary Information

Supplementary Legends.

Supplementary Figure S1.

Supplementary Figure S2.

Supplementary Figure S3.

Supplementary Figure S4.

Supplementary Figure S5.

Supplementary Table S1.

Supplementary Table S2.

## Data Availability

The datasets used and analyzed during the current study are available from the corresponding author upon reasonable request. The RNA-seq data of this study have been deposited in the Gene Expression Omnibus (GEO) under accession code GSE116530^16^.
