# Supplementary material for: Interlukin-4 weakens resistance to stress injury and megakaryocytic differentiation of hematopoietic stem cells by inhibiting Psmd13 expression
Source: Sci Rep. 2023 Aug 31;13:14253. doi: 10.1038/s41598-023-41479-6 (PMC10471741; doi:10.1038/s41598-023-41479-6)
Supplement: Supplementary file 1 — Supplementary Legends. [file 41598_2023_41479_MOESM1_ESM.docx]

**Supplemental Figure Legends**

**Figure S1. Cell cycle analysis of LKS+ cells in response to IL-4**

(A-B) Flow plots (A) and histogram (B) show the Ki67 staining of LKS+ cells in the absence or presence of mIL-4 (10ng/ml) for 24h.

(C) Absolute numbers of LKS+ cells in the absence or presence of mIL-4 (10ng/ml) after 24h.

Data are shown as mean ±SD. *p < 0.05, **p < 0.01, ***p < 0.001. ns, no significance.

**Figure S2. Representative FACS profiles of IL-4Rα^high^ and IL-4Rα^low^ LT-HSCs**

(A) Representative gating strategy for FACS sorting of IL-4Rα^high^ and IL-4Rα^low^ LT-HSCs for single cell colony, transplantation and tunicamycin assay.

(B) Representative gating strategy for apoptosis analysis of IL-4Rα^high^ and IL-4Rα^low^ LT-HSCs after 2Gy irradiation.

**Figure S3. Differential gene expression of LKS+ cells treated with IL-4 or not.**

1. Heatmap of gene expression in control and IL-4 treated LKS+ cells. The color scale indicates expression values.
2. Volcano plot of differentially expressed genes between control and IL-4 treated LKS+ cells. The X-axis represents the log2 fold change of gene expression levels. The Y-axis represents the –log10 P-value. Significantly upregulated genes are represented as ‘red’ dots and significant downregulated genes are represented as ‘green’ dots.
3. Negative enrichment of hematopoietic stem progenitor cell differentiation genes and megkaryocyte differentiation genes in LKS+ cells treated with IL-4; positive enrichment of apoptosis-associated genes and response to endoplasmic reticulum stress genes in LKS+ cells treated with IL-4.

**Figure S4. *Psmd13* knockdown assays**

(A) Representative FACS plots of EGFP+ LKS+ cells transduced by *Psmd13* shRNA (1#, 2# and 3#) or scramble control.

(B) qRT-PCR analysis of the relative expression of *Psmd13* in LKS+ cells transduced with three *Psmd13* shRNAs or a scramble contrl shRNA. 1# and 3# *Psmd13* shRNA with higher knockdown efficacy were selected for subsequent transplantation experiments.

(C) Representative FACS plots of peripheral blood platelet reconstituted by EGFP+ LKS+ cells transduced by *Psmd13* shRNA (1# and 3#) or scramble control.

(D-E) Reconstitution status of *Psmd13* shRNA (1# and 3#) or scramble control transduced LKS+ cells 4 weeks after transplantation. D, percentage of donor-derived EGFP+ cells in peripheral blood platelet. E, fractions of EGFP+ PB nucleated cells.

Data are shown as mean ±SD. *p < 0.05, **p < 0.01, ***p < 0.001. ns, no significance.

**Figure S5. Apoptotic analysis of *Psmd13* knockdown cells.**

1. Representative flow graphs show the apoptosis rates 72 hours after *Psmd13* shRNA (1# and 3#) or scramble control transfection of LKS+ cells.
2. Apoptosis rates of *Psmd13* shRNA (1# and 3#) or scramble control transduced LKS+ cells.
3. Flow cytometry of apoptosis analysis of bone marrow donor-derived LKS+ cells at 4 weeks after *Psmd13* KD LKS+ cell transplantation.
4. Apoptotic analysis of bone marrow donor-derived LKS+ cells at 4 weeks after *Psmd13* KD LKS+ cells transplantation.

Data are shown as mean ±SD. *p < 0.05, **p < 0.01, ***p < 0.001. ns, no significance.

**Table S1. Antibodies used for flow cytometry**

**Table S2. Ingenuity Pathway Analysis (IPA) of canonical pathways with significant difference between IL-4Ra^high^ and IL-4Ra^low^ HSCs**
