# Supplementary figures and images for: Interlukin-4 weakens resistance to stress injury and megakaryocytic differentiation of hematopoietic stem cells by inhibiting Psmd13 expression
Source: Sci Rep. 2023 Aug 31;13:14253. doi: 10.1038/s41598-023-41479-6 (PMC10471741; doi:10.1038/s41598-023-41479-6)

Figure S1. Cell cycle analysis of LKS+ cells in response to IL-4.

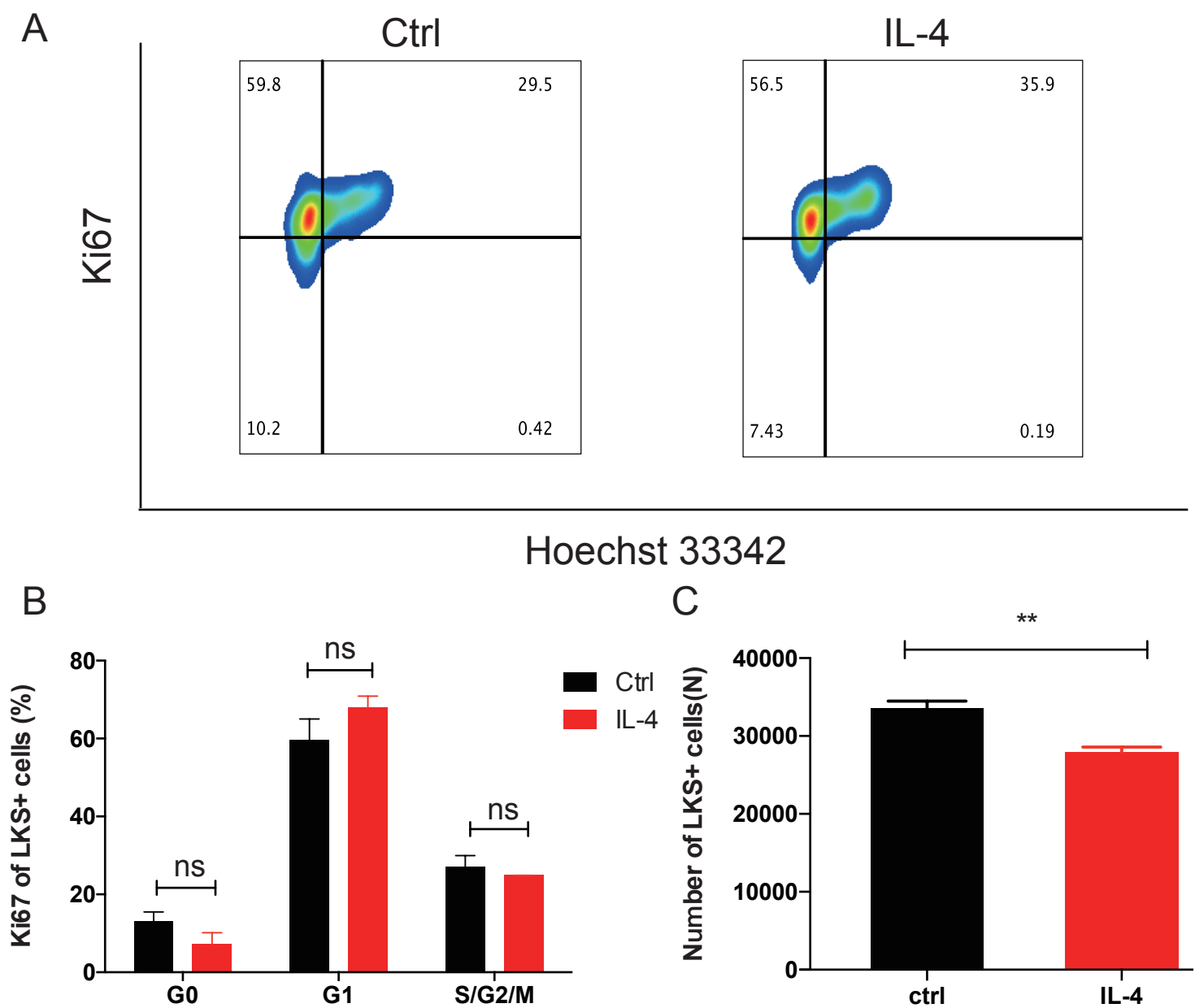

Supplement: Supplementary file 2 — Supplementary Figure S1. [file 41598_2023_41479_MOESM2_ESM.pdf]

Figure S3. Differential gene expression of LKS+ cells treated with IL-4 or not.

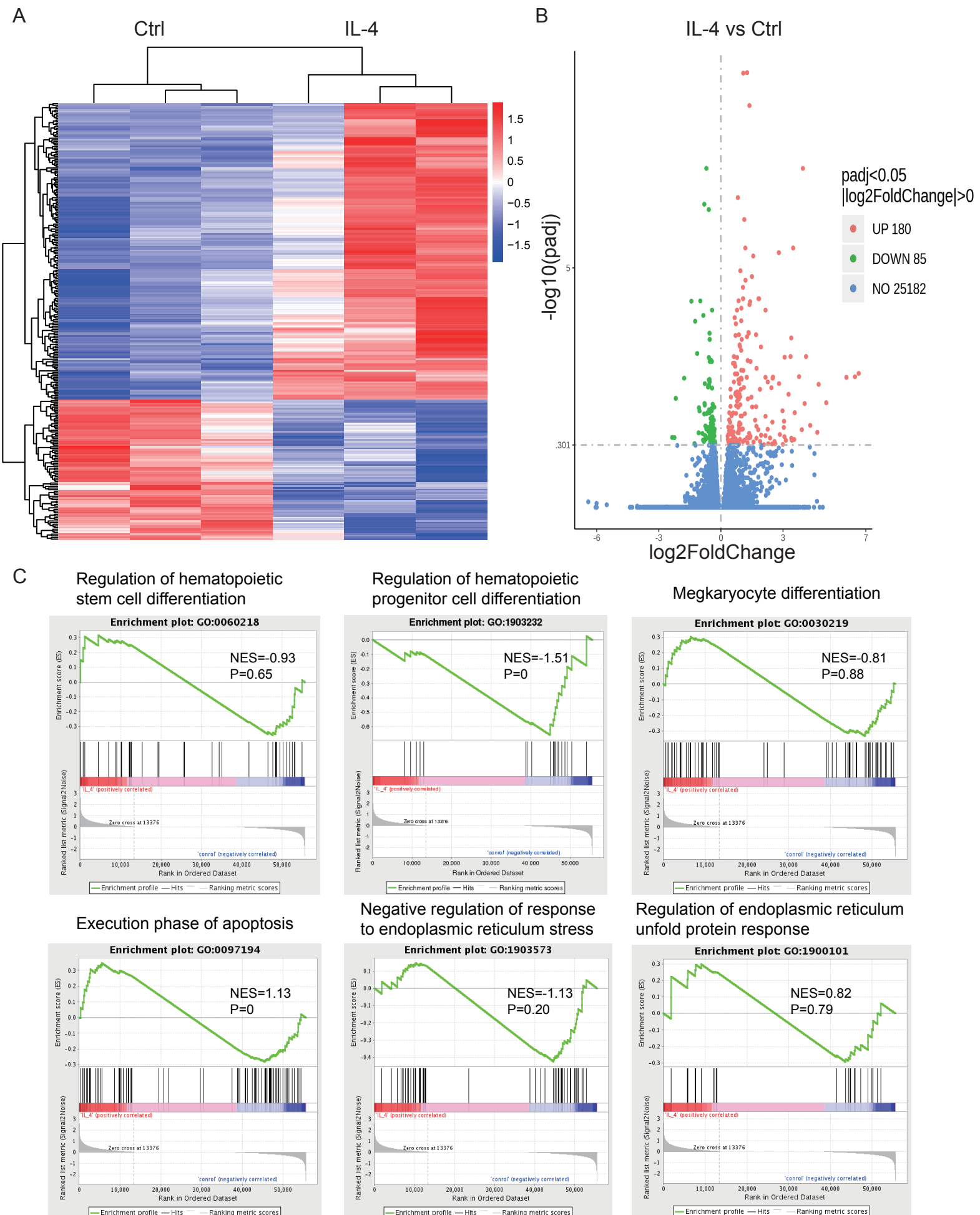

Supplement: Supplementary file 4 — Supplementary Figure S3. [file 41598_2023_41479_MOESM4_ESM.pdf]

Figure S4. Apoptotic analysis of Psmd13 knockdown cells.

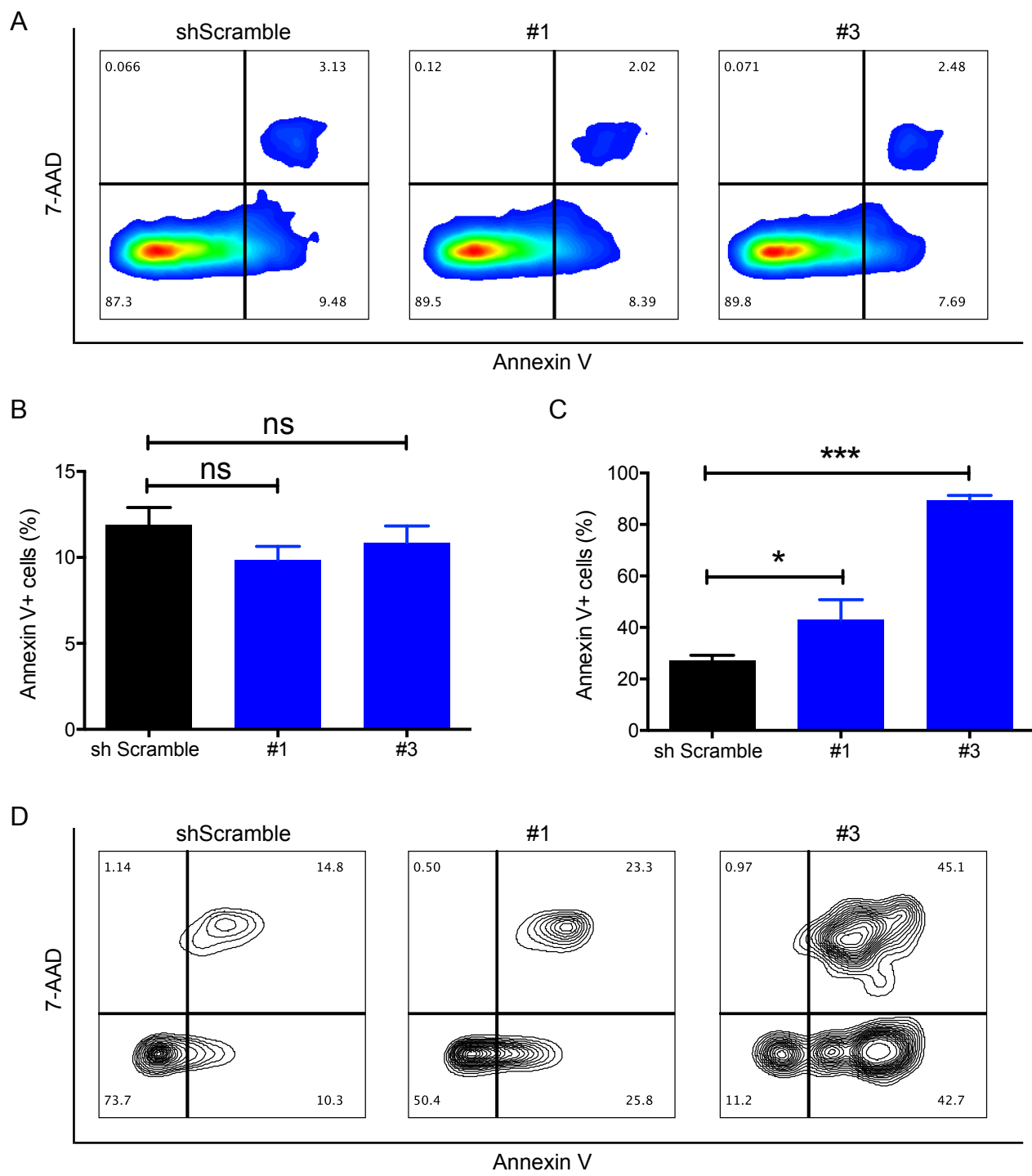

Supplement: Supplementary file 5 — Supplementary Figure S4. [file 41598_2023_41479_MOESM5_ESM.pdf]
