# Supplementary material for: Interlukin-4 weakens resistance to stress injury and megakaryocytic differentiation of hematopoietic stem cells by inhibiting Psmd13 expression
Source: Sci Rep. 2023 Aug 31;13:14253. doi: 10.1038/s41598-023-41479-6 (PMC10471741; doi:10.1038/s41598-023-41479-6)
Supplement: Supplementary file 3 — Supplementary Figure S2. [file 41598_2023_41479_MOESM3_ESM.pdf]

Figure S2. Representative FACS profiles of IL-4Rα^{high} and IL-4Rα^{low} LT-HSCs.

A

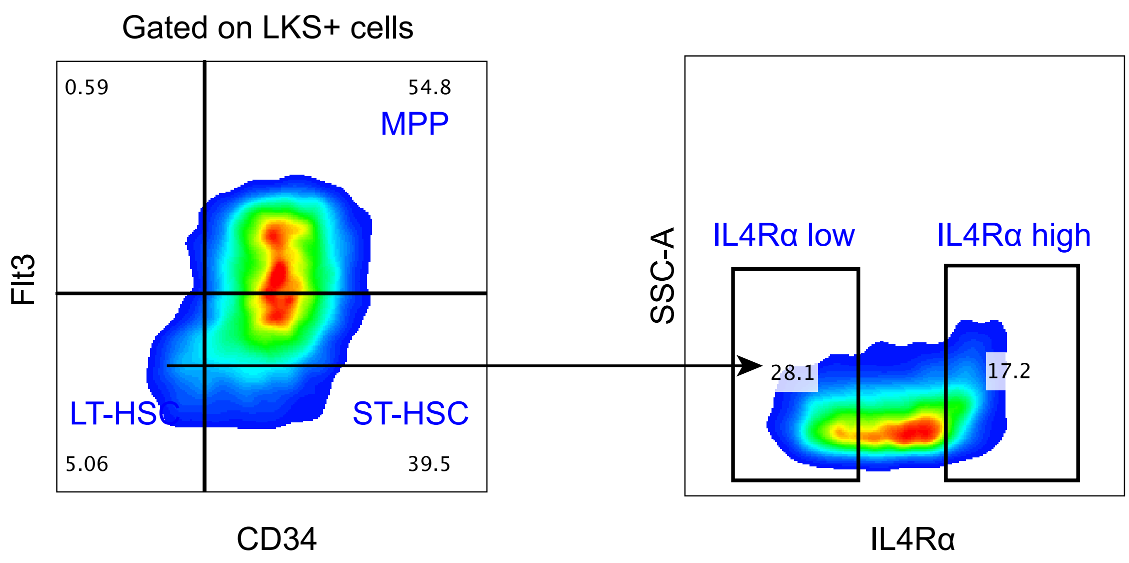

B

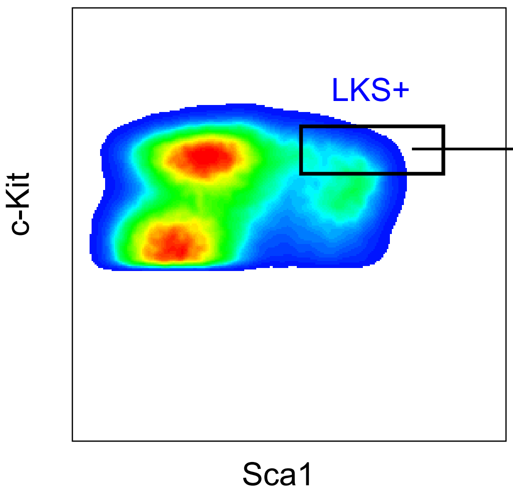

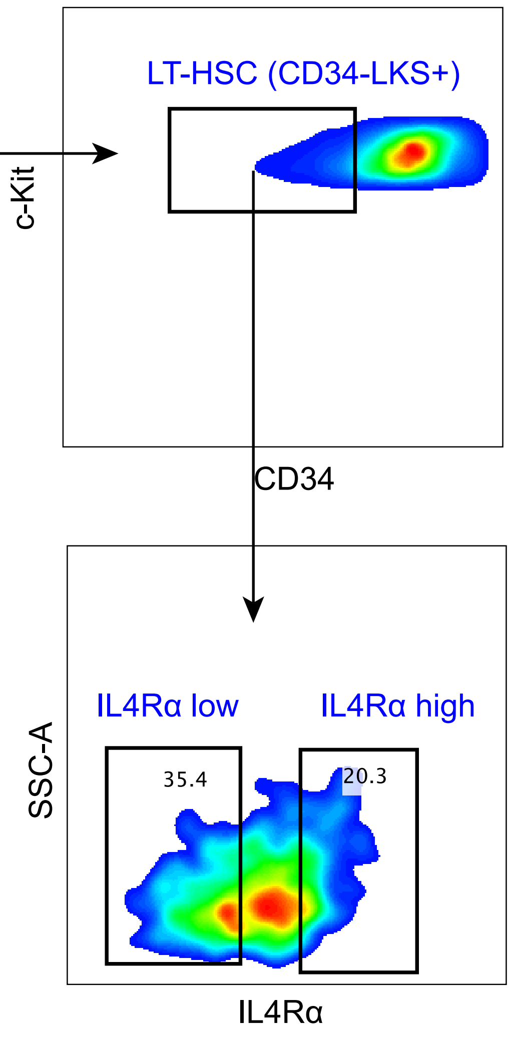

gating strategies for
A: purification of IL4Ra high vs low LT-HSC
for Tunicamycin assay
B: apoptosis analysis of IL4Ra high vs low LT-HSC
post irradiation
