# Supplementary material for: Interlukin-4 weakens resistance to stress injury and megakaryocytic differentiation of hematopoietic stem cells by inhibiting Psmd13 expression
Source: Sci Rep. 2023 Aug 31;13:14253. doi: 10.1038/s41598-023-41479-6 (PMC10471741; doi:10.1038/s41598-023-41479-6)
Supplement: Supplementary file 6 — Supplementary Figure S5. [file 41598_2023_41479_MOESM6_ESM.pdf]

Figure S5. Apoptotic analysis of Psmd13 knockdown cells.

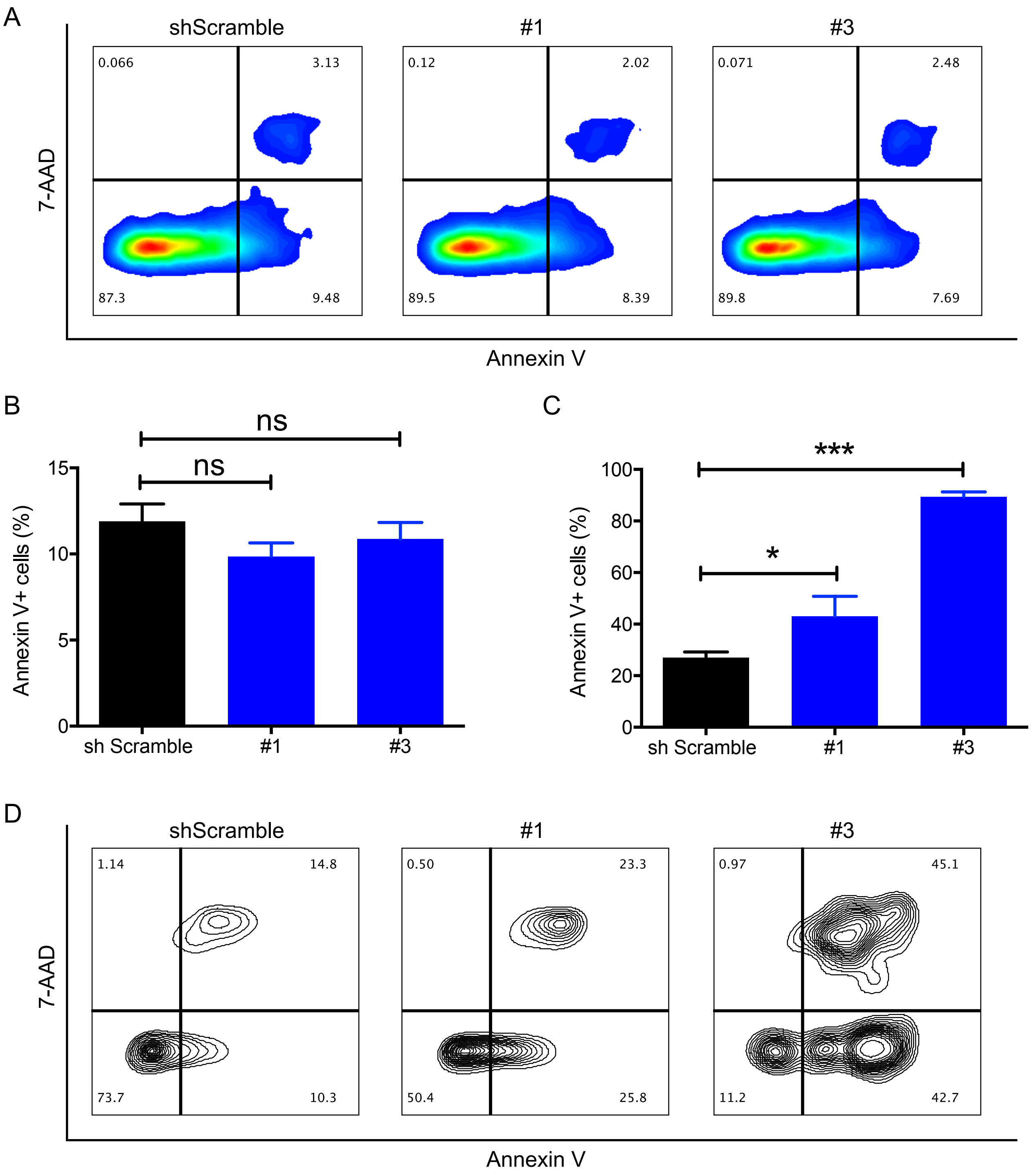

S5. Apoptotic analysis of Psmd13 knockd
