# Supplementary material for: Interlukin-4 weakens resistance to stress injury and megakaryocytic differentiation of hematopoietic stem cells by inhibiting Psmd13 expression
Source: Sci Rep. 2023 Aug 31;13:14253. doi: 10.1038/s41598-023-41479-6 (PMC10471741; doi:10.1038/s41598-023-41479-6)
Supplement: Supplementary file 7 — Supplementary Table S1. [file 41598_2023_41479_MOESM7_ESM.pdf]

Supplementary Table S1. Antibodies used for flow cytometry

Antibody conjugate	Clone	Supplier
B220 PE-CY7	RA3-6B2	eBioscience
B220 Biotin	RA3-6B2	eBioscience
CD3e Biotin	145-2C11	eBioscience
CD3e PE-CY7	145-2C11	eBioscience
CD4 Biotin	RM4-5	eBioscience
CD4 PE-CY7	RM4-5	eBioscience
CD8a Biotin	53-6.7	eBioscience
CD8a PE-CY7	53-6.7	eBioscience
Ter-119 Biotin	Ter119	eBioscience
Ter-119 PE-CY7	Ter119	eBioscience
Ter-119 PE	Ter119	eBioscience
Mac-1 Biotin	M1/70	eBioscience
Mac-1 PE-CY7	M1/70	eBioscience
Mac-1 APC-eFluor 780	M1/70	eBioscience
Gr-1 (Ly-6G) Biotin	RB6-8C5	eBioscience
Gr-1 (Ly-6G) PE-CY7	RB6-8C5	eBioscience
Gr-1(Ly-6G) APC-eFluor 780	RB6-8C5	eBioscience
Streptavidin APC-eFluor 780	-	eBioscience
Streptavidin BrilliantViolet 421	-	BioLegend
c-Kit (CD117) APC	2B8	eBioscience
CD34 Biotin	RAM34	eBioscience
CD34 FITC	RAM34	eBioscience
CD41 APC	MWReg30	eBioscience
CD41 PerCP-eFluor 710	MWReg30	eBioscience
Flt3 (CD135) PE	A2F10	eBioscience
Sca-1 PE-Cy7	D7	eBioscience
Sca-1 APC-Cy7	D7	BioLegend
CD124 BrilliantViolet 421	mIL-4R-M1	BD Bioscience
CD16/32 PE	93	eBioscience
CD150 PE	TC15-12F12.2	BioLegend
CD45.1 PE-CY7	A20	eBioscience
CD45 FITC	30-F11	eBioscience
Annexin V FITC	-	BD Bioscience
