# Supplementary material for: Interlukin-4 weakens resistance to stress injury and megakaryocytic differentiation of hematopoietic stem cells by inhibiting Psmd13 expression
Source: Sci Rep. 2023 Aug 31;13:14253. doi: 10.1038/s41598-023-41479-6 (PMC10471741; doi:10.1038/s41598-023-41479-6)
Supplement: Supplementary file 8 — Supplementary Table S2. [file 41598_2023_41479_MOESM8_ESM.pdf]

Supplementary Table S2. Ingenuity Pathway Analysis (IPA) of canonical pathways with significant difference between IL-4Rα^{high} and IL-4Rα^{low} HSCs

Ingenuity Canonical Pathways	-log(p-value)	Ratio	z-score
NRF2-mediated Oxidative Stress Response	3.92	0.115	0.632
Apoptosis Signaling	2.96	0.133	0.302
FAK Signaling	2.64	0.122	#NUM!
TNFR2 Signaling	2.6	0.2	-0.447
Angiopoietin Signaling	2.47	0.13	0.707
Chronic Myeloid Leukemia Signaling	2.39	0.114	#NUM!
Gα12/13 Signaling	2.31	0.104	-0.577
Renal Cell Carcinoma Signaling	2.27	0.122	-0.378
IL-3 Signaling	2.24	0.12	-0.632
Amyotrophic Lateral Sclerosis Signaling	2.2	0.108	#NUM!
Ketolysis	2.14	0.333	#NUM!
Protein Ubiquitination Pathway	2.1	0.083	#NUM!
Cholecystokinin/Gastrin-mediated Signaling	2.08	0.109	-0.302
Valine Degradation I	2.04	0.222	#NUM!
Ketogenesis	2	0.3	#NUM!
Role of NFAT in Cardiac Hypertrophy	2	0.0885	#NUM!
Molecular Mechanisms of Cancer	1.91	0.074	#NUM!
CD40 Signaling	1.91	0.114	0
Acute Myeloid Leukemia Signaling	1.9	0.108	-0.333
Methylmalonyl Pathway	1.9	0.5	#NUM!
Inflammasome pathway	1.88	0.2	0
Macropinocytosis Signaling	1.84	0.111	0
Telomerase Signaling	1.82	0.1	-0.816
PI3K/AKT Signaling	1.8	0.096	-0.632
IL-10 Signaling	1.79	0.116	#NUM!
Role of IL-17A in Arthritis	1.79	0.116	#NUM!
Assembly of RNA Polymerase III Complex	1.77	0.25	#NUM!
Regulation of eIF4 and p70S6K Signaling	1.76	0.0892	-0.378
IL-6 Signaling	1.73	0.0938	-1.155
B Cell Receptor Signaling	1.7	0.0838	-0.258
PAK Signaling	1.7	0.1	-1.265
Creatine-phosphate Biosynthesis	1.69	0.4	#NUM!
2-oxobutanoate Degradation I	1.69	0.4	#NUM!
Mevalonate Pathway I	1.67	0.231	#NUM!
LPS-stimulated MAPK Signaling	1.66	0.103	-1
VEGF Signaling	1.64	0.098	0
TWEAK Signaling	1.63	0.143	0.447
CXCR4 Signaling	1.61	0.0854	-0.302
Tryptophan Degradation III (Eukaryotic)	1.6	0.167	#NUM!
DNA Double-Strand Break Repair by Non-Homologous End Joining	1.58	0.214	#NUM!

Isoleucine Degradation I	1.58	0.214	#NUM!
Role of Pattern Recognition Receptors in Recognition of Bacteria and Viruses	1.55	0.0882	-1.414
FGF Signaling	1.55	0.0989	0.333
Chondroitin and Dermatan Biosynthesis	1.53	0.333	#NUM!
mTOR Signaling	1.52	0.0796	-0.302
Telomere Extension by Telomerase	1.5	0.2	#NUM!
Glutaryl-CoA Degradation	1.5	0.2	#NUM!
Death Receptor Signaling	1.49	0.0968	2.333
Thrombopoietin Signaling	1.47	0.108	-0.378
Cell Cycle: G1/S Checkpoint Regulation	1.47	0.108	0.447
Insulin Receptor Signaling	1.44	0.0851	-1.732
Chondroitin Sulfate Degradation (Metazoa)	1.43	0.188	#NUM!
Erythropoietin Signaling	1.42	0.0988	#NUM!
Neuroinflammation Signaling Pathway	1.42	0.0712	-0.229
Hereditary Breast Cancer Signaling	1.4	0.0839	#NUM!
Prostate Cancer Signaling	1.39	0.0928	#NUM!
Role of Macrophages, Fibroblasts and Endothelial Cells in Rheumatoid Arthritis	1.39	0.0707	#NUM!
PPARα/RXRα Activation	1.39	0.0795	1.667
Melanocyte Development and Pigmentation Signaling	1.37	0.0918	0.707
Leukocyte Extravasation Signaling	1.36	0.0758	0.277
Osteoarthritis Pathway	1.36	0.0758	-0.577
Superpathway of Geranylgeranyldiphosphate Biosynthesis I (via Mevalonate)	1.36	0.176	#NUM!
PI3K Signaling in B Lymphocytes	1.35	0.0846	-1
HGF Signaling	1.33	0.087	-0.333
IL-8 Signaling	1.33	0.0765	-0.535
